# A Sensor-Based Decision Support System for Transfemoral Socket Rectification

**DOI:** 10.3390/s21113743

**Published:** 2021-05-28

**Authors:** Michalis Karamousadakis, Antonis Porichis, Suranjan Ottikkutti, DeJiu Chen, Panagiotis Vartholomeos

**Affiliations:** 1 TWI-Hellas, 152 32 Halandri, Greece; michalis.karamousadakis@twi.gr (M.K.); antonis.porichis@twi.gr (A.P.); 2 KTH Royal Institute of Technology, 10044 Stockholm, Sweden; suranjan@kth.se (S.O.); chen@md.kth.se (D.C.)

**Keywords:** transfemoral, prosthesis, socket rectification, pressure sensors, fuzzy-logic inference engine, FEA

## Abstract

A decision support system (DSS) was developed that outputs suggestions for socket-rectification actions to the prosthetist, aiming at improving the fitness of transfemoral prosthetic socket design and reducing the time needed for the final socket design. For this purpose, the DSS employs a fuzzy-logic inference engine (IE) which combines a set of rectification rules with pressure measurements generated by sensors embedded in the socket, for deciding the rectification actions. The latter is then processed by an algorithm that receives, manipulates and modifies a 3D digital socket model as a triangle mesh formatted inside an STL file. The DSS results were validated and tested in an FEA simulation environment, by simulating and comparing the donning process among a good-fitting socket, a loose socket (poor-fit) and several rectified sockets produced by the proposed DSS. The simulation results indicate that volume reduction improves the pressure distribution over the stump. However, as the intensity of socket rectification increases, i.e., as volume reduction increases, high pressures appear in other parts of the socket which generate discomfort. Therefore, a trade-off is required between the amount of rectification and the balance of the pressure distributions experienced at the stump.

## 1. Introduction

The basic requirements for acceptance of a prosthesis by the patient are comfort, function and appearance [1]. Prosthetic devices that fulfill the requirements allow an amputee to walk relatively normally; i.e., the gait should resemble that of a healthy person. The requirements are mutually interrelated and the degrees of attainment of one requirement is influenced by the degree of fulfillment for the others. Prosthetic devices that are dysfunctional will result in friction, instability and pistoning, and therefore in discomfort due to excessive pressures exerted in specific regions of the stump [1,2]. Patients that experience serious discomfort because of pain and skin irritation, or instability during walking, will minimize the discomfort at the expense of functionality by adapting their gait [1,2]. Patients whose prosthesis does not meet the three requirements eventually abandon the prosthetic device [3].

Currently, the socket fitness assessment relies on visual inspection of gait performance and stump’s skin irritation, and on verbal feedback about the comfort experienced by the patient. The rectification actions, i.e., the socket shape adjustments for improving fitness, are dictated by a theoretical framework, proposed by Radcliffe and other pioneers [1,4], based on classic engineering mechanics principles and clinical observations [5]. The lack of quantification is a principal factor restricting the levels of understanding and knowledge in prosthetic biomechanics [6,7,8,9]. The qualitative approach does not provide detailed knowledge of the interface loading and requires very strong clinical experience to pick up the subtle indicators of poor fitness [7,10]. Wrongly distributed interface pressures or poor coupling of the stump and socket which has not been detected by the prosthetists might manifest themselves through skin breakdown several days or weeks after the socket is completed.

The qualitative and approximate considerations of the stresses exerted on the stump–socket interface often lead to trial and error decisions and multiple rectification iterations. As a result, the patient is required to visit a clinic multiple times over one or more weeks for finalizing socket rectification. Hence, there is a need for novel design tools that make use of quantitative information about the state of a residual limp during gait and employ digital technology to expedite the rectification procedure and provide superior results. To this end, several research groups have developed computer-aided methods for socket redesign. Some computer-aided socket design software for transfemoral amputees was developed which acts as a digital design tool operated by the prosthetist. The key elements of the digital design tool are digital functionalities of sculpting and patching the socket, together with the graphical modeling procedure; however, there is no decision support system and it does not make use of sensor information [11]. More recent work presented a virtual laboratory called the Virtual Socket Laboratory, where the user has at her/his disposal virtual tools that permit him/her to emulate the procedures applied by orthopaedic technicians during the traditional socket manufacturing, and in addition provides design decision assistance; however, there is no sensor-based quantitative information involved in the decision process [12]. The aforementioned work was fostered with shape optimization techniques to automate the search for a design solution [13]. To this end, a physics-based model of socket-stump assembly (simulating the pressure distribution during the donning process) is built, and then the optimization process within the computer-aided environment adopted for simulations is planned. A modeling–simulation analysis workflow has been developed that provides insights about the stump–socket interaction using patient specific data [14]. The main focus of that work was the detailed investigation of socket-stump interaction. To this end, it generates a detailed, subject-specific, three-dimensional finite element model of an entire residual limb from diffusion tensor MRI images. To complete the modeling–simulation–analysis workflow, the generated subject-specific residual limb model was integrated within an implicit dynamic FE simulation of bipedal stance to predict the potential sites of deep tissue injury. The approach is strictly computational: there is no experimental component or sensor information about the actual pressure distribution over the stump used. To the extent of the authors knowledge, none of the aforementioned works used pressure measurements from the socket–stump interface.

This paper presents a novel socket rectification tool that functions as a decision support system (DSS), and is used to support the course of action for achieving superior check-socket rectifications. The core algorithm is a fuzzy-logic inference engine (IE) [15], which uses experts’ rules in combination with quantitative information generated by pressure sensors, embedded in the socket, and outputs a set of rectification actions, i.e., changes of the geometry of the digital socket model. The latter are then processed by an algorithm which receives, manipulates and modifies the 3D digital socket model as a triangle mesh formatted inside an STL file. The verification of the inference engine and of the proposed rectification outputs is demonstrated using FEM simulations.

The paper contains the following Sections. In Section 2, the authors provide a description of the DSS concept and architecture. Section 3 presents the main blocks of the rule-based fuzzy inference system. Section 4 presents the operation and indicative results of the proposed DSS. In Section 5, DSS algorithm verification is demonstrated using FEM simulations. The paper concludes in Section 6 followed by the references.

## 2. Socketsense DSS Concept

The ultimate goal of SocketSense DSS is to benefit the patient in two ways: (1) the socket rectifications will lead to superior fitness, (assessed by comfort levels) compared to rectifications without any sensor measurements available, and (2) the socket rectification will be accomplished faster compared to the case where there is no SocketSense software support. The first steps towards the final goal, as presented in this paper, are the development of the DSS algorithm and its validation in simulation scenarios.

### 2.1. Description of Socketsense Sensor Configuration

**Figure 1:**
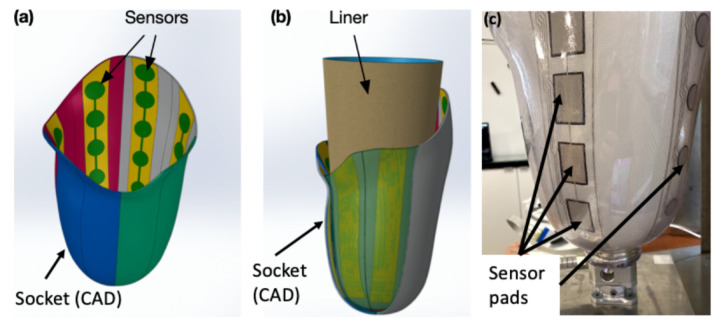
Socket with sensors: (**a**) CAD of sensors on the inner wall; (**b**) CAD of liner with socket; (**c**) actual socket with SocketSense sensors.

SocketSense technology is centered on a set of sensors which are firmly attached on the inner wall of the patient’s socket, as illustrated in Figure 1, and which measure pressure and shear forces.

**Figure 2:**
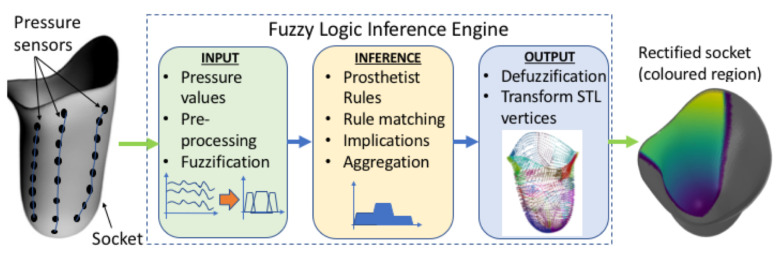
Main blocks of the DSS process.

The SocketSense system works with a patient’s existing socket or a patient’s check-socket (i.e., a diagnostic socket worn before producing the definitive socket). The sensor strips and/or shear sensors are firmly attached (taped) on the inner surface of the patient’s socket, covering important anatomical landmarks or known pressure-sensitive areas. The liner that will be worn by the patient acts as a physical interface (layer) between the residual tissue and the sensors (see Figure 1b). The socket has been divided into anatomical regions according to the segmentation and annotation proposed in [10]. These divisions are projected on the 3D surface of the socket and each of the regions might contain multiple, one or no sensor pads. The pressure measurements of the sensor pads at each region are averaged to give a single mean value. This translates to about 20–25 average measurements which constitute the input to the system, as shown in Figure 2. The number of input values is not constant, since it depends on the deployment of the sensors on the various anatomical regions of the socket. An indicative example of sensor deployment in the socket is presented in Table A2, in the Appendix A.

The neck of the sensor extends out of the socket and is connected to an embedded electronics system mounted on the outer wall of the socket, which performs data acquisition, processing and communication. The sensor measurements are collected real-time and transmitted wirelessly to a PC or tablet that runs the SocketSense software application. The spatial and temporal variations of the pressures over the residual limp allow the prosthetist to monitor the performance of the patient’s existing socket. These pressure data are used as input by the SocketSense DSS algorithms, presented in the next paragraph, to redesign the socket and improve its fitness.

### 2.2. DSS Architecture

The authors opted to develop the DSS based on a fuzzy inference system, although alternative approaches such as genetic algorithms and neural networks can perform just as well in many cases. Expert systems, and in particular, fuzzy logic have some properties that render them more suitable for the SocketSense DSS, as explained next. Deep learning approaches require vast amounts of data. These data for transfemoral amputees do not exist and would be very costly and time consuming to generate them, since such data would require a large number of amputees. On the other hand, a fuzzy-logic approach based on expert-knowledge and reasoning has the advantage that the solution to the problem can be cast in terms that human operators can understand, so that their experience can be used in the design of the inference engine. This makes it easier to mechanize tasks that are already successfully performed by humans, such as the actions for improvement of the socket design. The use of the sensor measurements provides data about the state of the stump–socket interface which can be processed by a computational algorithm such as the FIS. The architecture of the DSS system is depicted in Figure 2.

It comprises three blocks. The first is the input block responsible for receiving and fuzzifying the pressure time-series. The second is the inference engine block which combines a rule base with logical inference to conclude the rectification action. The third is the output block which defuzzifies the rectification actions into crisp values that dictate the socket shape modifications. The fuzzy membership functions, the rule base and the inference engine are described in the next two Sections.

## 3. Fuzzy Membership Functions and Rule Base

The current practice of socket rectification relies on a set of rectification rules and on the prosthetist’s subjective evaluations about the socket’s fitness. The rules are based on the underlying mechanics of the stump–socket interface and on empirical knowledge. The rules express relationships of causality that relate the evaluations made by the prosthetist (the cause) to the rectification actions (the effect). The evaluations refer to the appraisal of the socket fitness and comfort made by the prosthetist after observing the stump–skin irritation and the socket firmness (by pushing the stump against the socket and measuring the gap), and after monitoring the gait pattern of the patient during motion. Therefore, evaluations are subjective and rough estimates based on qualitative information. The rectification actions are the volume changes of the socket, measured either by deforming the socket walls using a heat-gun or by adding silicon pads in the interior regions of the socket. The degree of rectification (for example, the thickness of the wall deformation or the thickness of the inserted silicon pad) is a coarse-grain decision made by the prosthetist. Both evaluations and rectifications are described by the prosthetist using linguistic descriptions—for example, evaluations are expressed as low-pressure at Scarpa’s triangle and high pressures at distal-end of femur, and socket rectification actions are expressed as small, medium or high socket-volume reduction.

The aforementioned description of socket rectification actions is seen as a process that accepts as input the fitness evaluations and is based on a set of rules that generates as output the socket rectifications. The input, the output and the set of rules can be represented by linguistic descriptions using appropriate linguistic variables that characterize the degree of fitness and the degree of socket shape modification. Hence, a fuzzy logic framework can be readily used to represent the linguistic variables as fuzzy membership functions and a set of rules as fuzzy logic statements which are processed by an inference engine to produce the rectification result. The process implemented as a fuzzy logic inference system (FIS) is described in the next three subsections.

### 3.1. Linguistic Variables as Fuzzy Membership Functions

A fuzzy variable can take values anywhere in the range from “absolutely false” to “absolutely true”; therefore, fuzzy logic represents a superset of the conventional “crisp” logic [16]. This notion of partial truth makes fuzzy logic a very good means to model the natural uncertainty of language used by the prosthetist. It is partially true that a pressure can be high or normal, and therefore a statement about pressure felt is uncertain. Even if a pressure is measured (as is done in SocketSense), how the pressure is felt and interpreted by the patient is uncertain. Additionally, there is uncertainty when the prosthetist expresses verbally the extent to which a socket is rectified, since it is very difficult to calculate the exact deformation for a socket.

**Figure 3:**
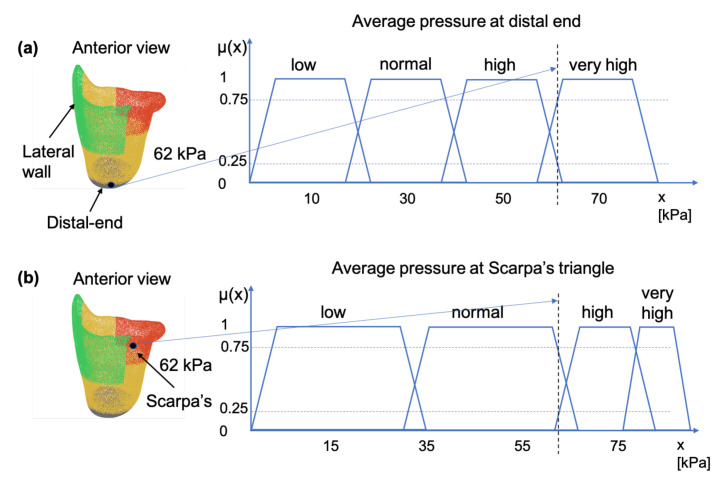
Membership functions for pressure measurements taken at: (**a**) the distal-end, and (**b**) at the Scarpa’s triangle. The two areas are indicated on the socket figure by the black dot.

The prosthetist uses linguistic variables for expressing pressure exerted on the socket. This variable may be assigned one or more linguistic values, which are in turn connected to a measured pressure value through the mechanism of membership functions. Figure 3, illustrates this concept by showing how a pressure measurement may be connected to the linguistic variable “pressure” through the membership functions $\mu_{i}\left(p\right)$ “low”, “normal”, “high” and “very high”, where subscript i={1,...,n} indicates the ith anatomical region of the socket. For example, for a sensor reading of 62 kPa, at a pressure sensitive area such as the distal-end, the linguistic variable pressure can be characterized as having 0.25 membership in the fuzzy set “high”; 0.75 membership in the fuzzy set “very high”; and 0.0 membership in the fuzzy set “normal”—as shown in Figure 3a. The same pressure value would have resulted in different characterizations if it were measured at Scarpa’s triangle, a region which is more pressure-tolerant than the distal-end region. For example, in the case of Scarpa’s region, as shown in Figure 3b, the sensor reading of 62 kP can be characterized as having 0.25 membership in the fuzzy set high, 0.75 membership in the fuzzy set normal and 0.0 membership in the fuzzy sets very high and low.

**Figure 4:**
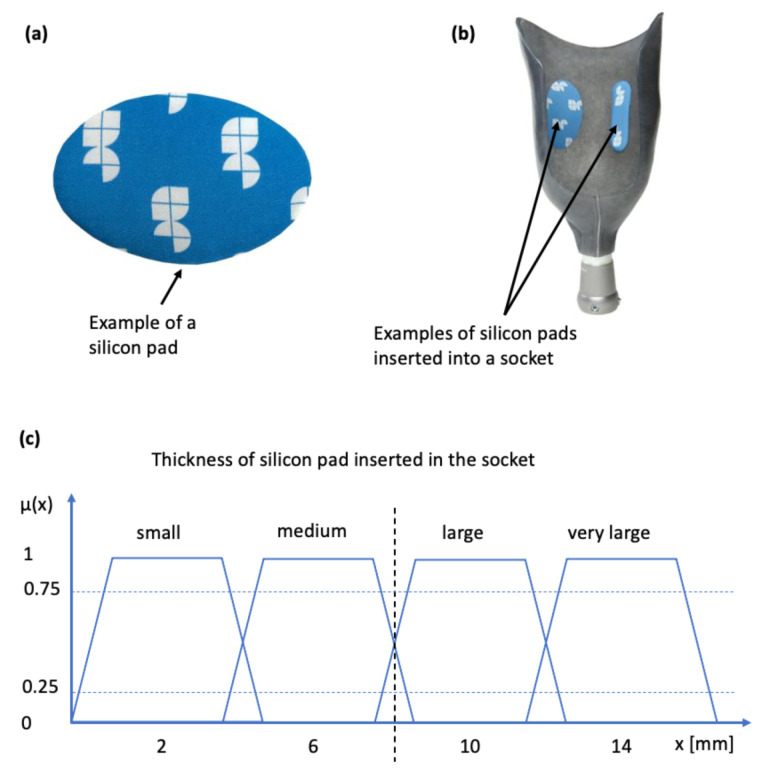
Silicon pads for socket rectification and associated membership function: (**a**) silicon pad; (**b**) silicon pad inserted into the inner wall of the socket (sticks due to adhesives); (**c**) membership function connecting the linguistic variable to pad thickness.

The case of socket rectification involves both crisp and fuzzy values. The specification of the socket area to be rectified does not involve any uncertainty, because it depends strictly on the anatomy of the stump which is well defined by the bony structures and other anatomical elements such as Scarpa’s triangle and the ischial tuberosity. The type of the rectification action also does not involve any uncertainty, it will be either the insertion of a silicon pad (as shown in Figure 4a,b) or a wall deformation using a heat-gun and manual shape deformation. However, the magnitude of the rectification (of any type) involves uncertainty because it is based on qualitative rules and empirical knowledge which are expressed by the prosthetist using natural language. The magnitude of the rectification is the thickness of the silicon pad, or the curvature of the deformed part of the socket wall. The degree of truth of the linguistic variable “rectification magnitude” is represented by the membership functions “small”, “medium”, “large” and “very large”. Specifically, in the case of the silicon pads, magnitude is connected to the the membership function μ(x) of thickness in mm, as indicated in Figure 4c. Hence, rectification for each anatomical region can be defined by the triple

(1) $$R_{i}=\left\{\mu \left(x\right),\left(r,\theta \right),\left\{pad,heatgun\right\}\right\}$$

where i={1,2,...,n} is the index of the anatomical region related to the rectification area, μ is the fuzzy variable of thickness, r,θ are the polar coordinates of the location on the socket where rectification will be applied and {pad,heatgun} is the type of the rectification (a categorical variable). The membership functions for the linguistic variables of pressure and rectification magnitude are used as the premises of the fuzzy logic sentences that formulate the rectification-rules, as described in the next paragraph.

#### Input Membership Function Normalisation

The linguistic variable “pressure” quantified by the membership functions $\mu_{i}\left(x\right)$ “low”, “normal”, “high” and “very high”, for each anatomical region *i*, is normalized with respect to pressure threshold values. Hence, the value of *x* is given as a percentage of pressure above a pressure threshold value. These thresholds are adjusted based on the load-tolerance ability of the residual limb of the patient. The load-tolerance ability can be found after performing tests with the patient as described in [17,18].

### 3.2. The Rule Base

**Figure 5:**
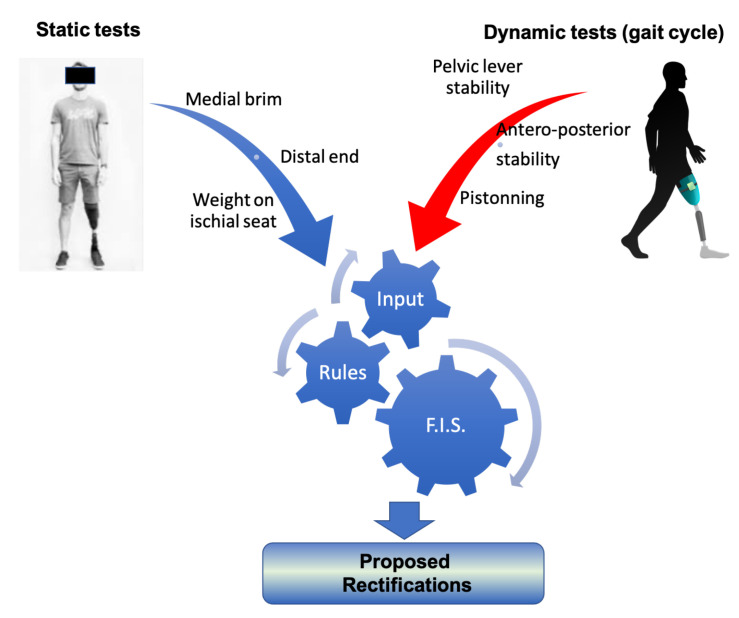
The classification of rectification rules.

A set of rules (a rule base) has been formulated that captures expert knowledge about how to rectify a prosthesis socket. The rules have as antecedents the linguistic variables of input pressure membership functions and as outputs the rectification magnitude membership functions and the specification of the region where rectification will take place. The rectification rules can be divided into two categories. The first are the static rules which incorporate pressure observations from static conditions, such as the patient standing straight (after the donning process); and the second are the dynamic rules which incorporate observations from dynamic conditions made during the phases of the gait cycle. For the former, it is necessary to know the pressures at the distal end of the stump, on the medial brim and on the ischial seat. For the latter, it is important to know the dynamic profile of the pressures related to the pelvic-lever stability, antero-posterior stability and pistoning during gaiting [1,2], as shown in Figure 5. Although, in the long term, the ultimate goal of this research is to develop a rule base that incorporates both static and dynamic cases, in the scope of this paper the rule base is limited to static rules, which is the first step towards the ultimate goal. The methodology presented in the following paragraphs is demonstrated for a transfemoral quadrilateral socket, but it can be applied to other type of sockets (ischial containment, etc.) and to other types of amputation.

In the static case, the prosthetist observes the static pressures on the stump by asking the patient to perform the donning of the socket and to stand straight for a short duration of time. Based on questionnaires, a verbal description and by visual inspection of the stump surface (looking for irritated regions) after doffing, the prosthetist follows a set of rules to make decisions on the rectification actions. The rectification rules are determined by studying the literature of transfemoral prosthesis and by interviewing prosthetists. Examples of rectification rules that are applied frequently are described next.

#### 3.2.1. Rule Description: The Distal-End of the Stump Should Not Experience High Pressures

**Figure 6:**
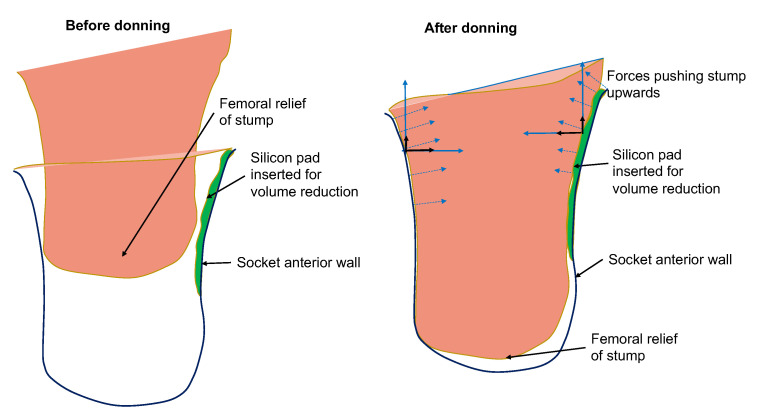
Illustration of rectification action based on silicon-pad insertion for avoiding the development of distal-end pressures. The forces developed by the silicon pad prevent the stump from sliding deep into the socket.

**Physical mechanism:** Any pressure against the stump’s distal-end tissues (femoral relief) should be of low magnitude to prevent the development of edema in the region. High pressures at the distal-end can occur due to two reasons: (a) the length of the socket is too short and exerts high pressures on the stump; (b) the socket is loose and as a result the stump slides down into the socket and pushes against the bottom of the socket. An indication that the socket is loose is given by low pressure readings at Scarpa’s triangle region. In the latter case, the rectification action is the reduction of volume of the socket by inserting a silicon pad on the proximal areas of the socket. The result of the rectification is a tighter socket that holds and fastens the stump tissue relatively high in the socket so that the distal-end does not push against the socket wall and no high pressures are exerted, as is demonstrated in Figure 6.

The above linguistic description is expressed using propositional logic and includes the following antecedents and consequences, where all variables are represented through the corresponding membership functions:

- Antecedents:
  – – *P*: High pressure at distal-end;
  – *Q*: Medium pressure at distal-end;
  – *M*: Low pressure at distal-end;
  – *N*: Low pressure at Scarpa’s triangle.
- Consequences:
  – *R*: Large volume reduction of anterior area at Scarpa’s area;
  – *S*: Medium volume reduction of anterior area at Scarpa’s area;
  – *T*: Low volume reduction of anterior area at Scarpa’s area;
  – *U*: Socket is too short and has to be replaced.
- Rule set I:
  (2) $$\begin{array}{c} P\wedge N\Rightarrow R \end{array}$$
  (3) $$\begin{array}{c} Q\wedge N\Rightarrow S \end{array}$$
  (4) $$\begin{array}{c} M\wedge N\Rightarrow T \end{array}$$
  (5) $$\begin{array}{c} P\wedge (\neg N)\Rightarrow U \end{array}$$

The rules form guidelines for the placement of the embedded sensors. For example, rules (2)–(5) indicate that sensor measurements are required at the anatomical regions of the distal-end (femoral relief of stump) and Scarpa’s triangle.

#### 3.2.2. Rule Description: Maintain Ischium in Place on the Ischial Seat

**Physical mechanism:** The socket, especially a ischial tuberosity (IT) socket, provides a definite ischial shelf to transmit the vertical load; this is called the ischial seat. If the ischial tuberosity is not properly seated on the ischial seat, high pressures may appear over the ischial and medial area which cannot be tolerated by the patient. If the socket is loose, the ischial tuberosity will slide inwards the socket, ride on the edge of the ischial shelf and wedge the stump into the anteromedial apex, generating high shear-forces on medial side and great discomfort to the patient [1]. To maintain the ischium in place properly, considerable counter pressure from the front of the socket is required. This is achieved by compressing the pressure tolerant soft tissues such as those of Scarpa’s triangle area along the anterior aspect of the stump, for example, by inserting a silicon pad of adjusted thickness over the anterior wall of the socket. The rules are expressed using propositional logic and include the following antecedents and consequences:

- Antecedents:
  – *P*: High pressures on pubic ramus on the medial wall of the socket;
  – *Q*: Low pressure at ischial seat;
  – *N*: Normal pressures at ischial seat.
- Consequences:
  – *R*: Insert silicon pad of high thickness over the anterior wall;
  – *S*: Insert silicon pad of medium thickness over the anterior wall.
- Rule set II:
  (6) $$\begin{array}{c} P\wedge Q\Rightarrow R \end{array}$$
  (7) $$\begin{array}{c} P\wedge N\Rightarrow S \end{array}$$

The rules form guidelines for the placement of the embedded sensors. For example, rules (6) and (7) indicate that sensor measurements are required at the anatomical regions of the pubic ramus and ischial seat.

#### 3.2.3. Rule Description: Achieve Proper Degree of Tightness of Fit along the Length of the Stump

**Physical mechanism:** The lateral wall should be shaped to fit the stump accurately, and should, if necessary, be flattened to distribute the lateral support pressure over a large area so that it can be tolerated comfortably. If the force distribution is not uniform enough or does not offer the proper degree of tightness of fit along the length of the stump, the insertion of a silicon pad along the lateral wall is required in order to reduce the medio-lateral dimensions and increase socket fitness. The rule for the lateral wall is expressed using propositional logic as:

- Antecedent:
  – *P*: Pressure along the lateral side of the stump is not uniform (especially mid and proximal regions);
  – *Q*: The pressure along the lateral side is low;
  – *R*: The pressure along the lateral side is medium.
- Consequence:
  – *T*: Socket should be rectified by inserting normal thickness silicon pad on the lateral side;
  – *U*: Socket should be rectified by inserting large thickness silicon pad on the lateral side.
- Rule set III:
  (8) $$\begin{array}{c} P\wedge Q\Rightarrow U \end{array}$$
  (9) $$\begin{array}{c} P\wedge R\Rightarrow T \end{array}$$

A standard fuzzy inference engine implemented in Python called scikit-fuzzy [19] is used to generate conclusions about rectifications. The inference engine determines which rules to trigger, computes the implication of each rule independently, aggregates the conclusions and defuzzifies the aggregated conclusion into a crisp value for socket thickness rectification at a particular area.

## 4. Rectification of the 3D Digital Model

In SocketSense, the outputs of the inference engine (i.e., the magnitudes of the rectification actions) need to be applied in an existing 3D digital socket model. Since the socket model contains a number of mesh elements, a subset of them needs to be deformed to a new location in space. Thus, there is a need for deformations that run locally and offer finer control over each element of the mesh. Hence, surface-based deformations are used.

### Deformation Algorithms

The deformation algorithms proposed in the literature use the following rationale [20]:

The deformation of a given surface *S* into the desired surface $S^{\prime }$ is described by a displacement function *d* that associates to each point p in *S* a displacement vector d(p). By this vector, it maps the given surface to its deformed version $S^{\prime }$:

(10) $$S^{\prime }:=p+d(p)\;|\;p\in S$$

The user though, controls the deformation by prescribing displacements $d_{i}$ for a set of so-called handle points $p_{i}$ in H⊂S, and by constraining certain parts F⊂S to stay fixed during the deformation:

(11) $$\begin{array}{cc} d(p_{i}) & =d_{i},\forall p_{i}\in H \end{array}$$

(12) $$\begin{array}{cc} d(p_{i}) & =0,\forall p_{i}\in F \end{array}$$

Additionally, there is an unconstrained deformation region R=S(H∪F) in which the points of the surface should be displaced but are not controlled by the user. All the deformation algorithms try to optimize the displacement vectors $d_{i}$ for all the remaining unconstrained vertices $p_{i}$ in *R*, such that the resulting shape deformation meets the user’s expectations.

The rationale followed by the developed surface-based deformation algorithm is slightly different than the one proposed in the literature. In SocketSense there are clearly only handle points set by the user implicitly (it is the knowledge of the prosthetist which determines the rule base). The output of the fuzzy inference system is the magnitude of inner volume reduction of the socket (i.e., increasing the thickness of the inner socket wall in specific regions). This translates to the requirement of specific displacements $d(p_{i})$ in specific wall regions (determined by the prosthetist’s experience).

**Figure 7:**
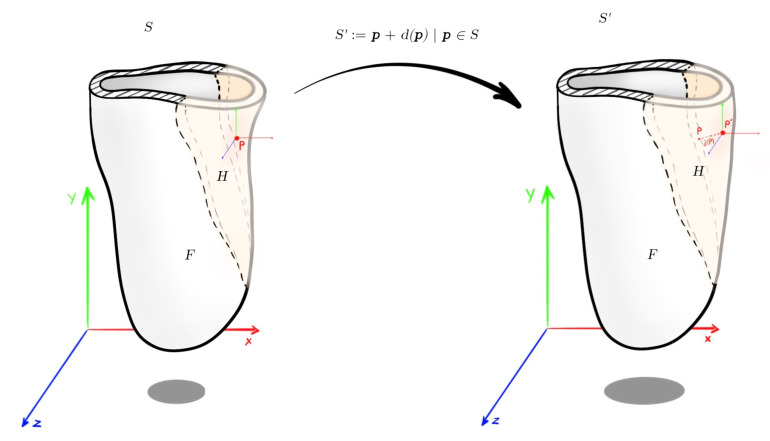
Socket deformation with the mathematical notation used.

As a result, the settings for the algorithms should be the same as before, but with the difference that there is no unconstrained deformation region *R*, only desired control region *H* and fixed region *F*. Therefore, no optimization needs to be run, such as solving a PDE or a linear system of equations. A useful illustration with the deformation notation is shown in Figure 7.

With that in mind, each point in the control region is displaced in the direction of its normal (in order to preserve geometric details under local rotation). For the implementation, an open-source python library called trimesh [21] is used. Most results were visualized with trimesh or MeshLab [22]. The normal displacement can have many forms. In the first iteration, the Algorithm 1 was used.

**Algorithm 1:** Constant value as normal displacement.**Result**: The rectified mesh $m^{\prime }$ **Input**: The current mesh *m*   The constant normal displacement C   The angle to start rectification $\theta_{start}$   The angle to finish rectification $\theta_{end};$ where $\theta_{end}>\theta_{start}$ 
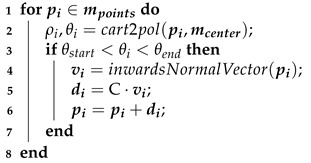


It should be noted that the *H* region is the inner surface area located between two given angles, $\theta_{start}$ and $\theta_{end}$. After consulting with a number of prosthetists, a modified version of Algorithm 1 was created for volume reduction on the antero-lateral wall of the socket. The modifications to the previous algorithm are the following:

- The edges of the region left-wise and right-wise (10%) are smoothed with a logistic function [23], so that there is a smooth transition between the fixed region and the controlled region.
- The two levels of the volume reduction (the FIS returns two outputs; reduction magnitude on anterior and lateral) are smoothed with a logistic function [23], so that there is a smooth transition between the two levels.
- The normal displacement of each point is normalized with respect to the maximum height found in the *H* region.

Thus, the final displacement $d_{i}$ is produced by $v_{i}$ (the normal vector of each point $p_{i}$) and multiplied by a variable $c_{i}$, instead of a constant value C. The variable $c_{i}$ is created with the following two steps. The first step is a piece-wise function and the second step is a normalization with respect to the maximum height found in the *H* region. The first step is represented mathematically as follows:

**Step 1:**

(13) $$c_{i}=\left\{\begin{array}{cc} 0, & \mathrm{if}\;p_{i}\in F.\\ f_{l}\left(\theta \left(p_{i}\right)\right), & \mathrm{if}\;\theta_{AS}\leq \theta \left(p_{i}\right)\leq \theta_{AS}+0.1\cdot \Delta \theta_{max}.\\ m_{A}, & \mathrm{if}\;\theta_{AS}+0.1\cdot \Delta \theta_{max}\leq \theta \left(p_{i}\right)\leq \theta_{AL}-\frac{\Delta \theta_{max}}{2}.\\ f_{l}\left(\theta \left(p_{i}\right)\right), & \mathrm{if}\;\theta_{AL}-\frac{\Delta \theta_{max}}{2}\leq \theta \left(p_{i}\right)\leq \theta_{AL}+\frac{\Delta \theta_{max}}{2}.\\ m_{L}, & \mathrm{if}\;\theta_{AL}+\frac{\Delta \theta_{max}}{2}\leq \theta \left(p_{i}\right)\leq \theta_{LE}-0.1\cdot \Delta \theta_{max}.\\ f_{l}\left(\theta \left(p_{i}\right)\right), & \mathrm{if}\;\theta_{LE}-0.1\cdot \Delta \theta_{max}\leq \theta \left(p_{i}\right)\leq \theta_{LE}. \end{array}\right.$$

where

- $f_{l}$ is the logistic function:
  (14) $$f_{l}\left(x\right)=\frac{L}{1+\exp(n\cdot k\left(x-x_{0}\right))}$$
  where
  – $x_{0}$, the *x* value of the sigmoid’s midpoint.
  – *L*, the curve’s maximum value.
  – *k*, the logistic growth rate or steepness of the curve.
  – *n*, the direction of the curve (up-down or down-up).
  The parameter values used for the logistic function are shown in Table A1.
- $\theta (p_{i})$ is the angle of the point $p_{i}$.
- $\theta_{AS}$ is the starting angle on the anterior wall.
- $\theta_{AL}$ is the middle angle on the antero-lateral wall.
- $\theta_{LE}$ is the end angle on the lateral wall.
- $m_{A}$ is the magnitude of the rectification on the anterior wall.
- $m_{L}$ is the magnitude of the rectification on the lateral wall.
- $\Delta \theta_{max}$ is the absolute maximum angle difference $|\theta_{AS}-\theta_{LE}|$.

**Step 2:**

(15) $$c_{i}=c_{i}\cdot \frac{h_{i}}{h_{max}}$$

where

- $h_{i}$ is the height of the point $p_{i}$.
- $h_{max}$ is the maximum height found in the *H* region.

**Figure 8:**
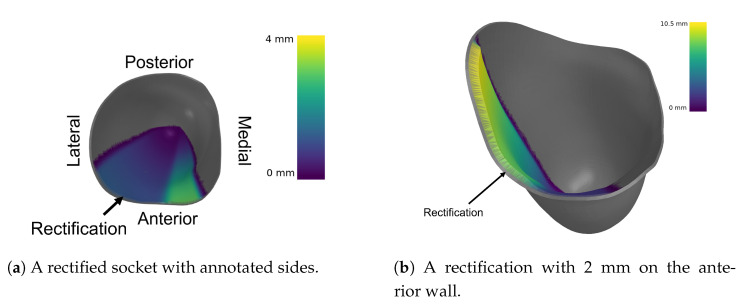

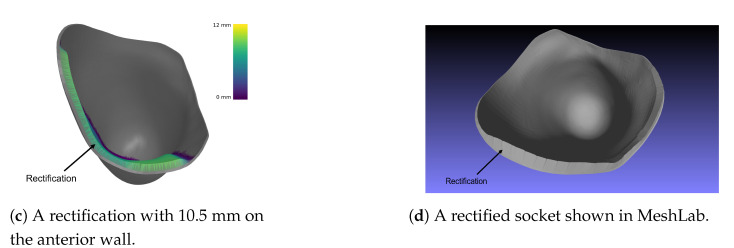
Different rectifications of an ischial containment socket produced by the DSS.

The results of said algorithm are shown in Figure 8.

## 5. Demonstration of Algorithmic Execution

A validation procedure was designed for demonstrating that the DSS generates rectification suggestions that lead to pressure distributions which improve the fitness and comfort of the socket. The validation procedure was based on a finite elements analysis (FEA) simulation, replicating the process of donning and predicting the pressure distribution on the stump.

### 5.1. Finite Elements Simulation Setup

The ANSYS Static structural analysis package was used to simulate the contact forces occurring between the socket and the stump during donning. The geometries of the socket and femur were acquired from thingiverse [24]. A left-side quadrilateral socket and femur were used. The interior surface of socket was used to generate a stump by closing and filling the surface at the proximal brim. A quasistatic simulation was conducted using a donning force of 50 N over one second, and subsequently a statically standing 100 Kg amputee piston force of 500 N over the next second. Material properties used for the bodies were identical those in a study by Lacroix [25]. The femur and socket were considered to be linearly elastic, whereas the stump followed a hyperelastic Mooney–Rivlin model, as described in Table 1. Rigid contact was assumed between the femur and stump, whereas frictional contact was defined between the socket and stump with a frictional coefficient of 0.415.

The simulation was repeated for a loose socket, implying the cross-sectional area of the socket was increased by 5%. The number of mesh elements varied between 60,000 and 70,000 based on the socket used. The numbers of elements and pressures produced by the FEA simulation were sufficient and well in range for models and results indicated by the comparison of several references in a systematic review conducted by A.S.Dickinson [26].

The FEA simulation generated a pressure distribution over the entire stump. However, in reality the DSS accepts pressure measurements generated by a number of sensors fixed on particular points on the socket. In order to simulate the localised sensor measurements, the pressure distributions at the locations of the sensors (defined by the actual socket) were averaged to form the DSS input values. These values were then provided to the DSS, which processed them and produced rectified sockets calibrated based on sensor pressures from nominal and loose sockets. The nominal socket was considered a benchmark of comfortable socket (set by the authors for demonstration purposes). The determination of the pressure thresholds, as described in Section 3, was performed based on the nominal socket pressure distribution. The loose socket was constructed to simulate a poor-fitting socket. The simulation steps were as follows. The nominal socket was simulated and the resulting pressure distribution was used to define pressure thresholds for normalization of the pressure measurements. Then the loose socket was simulated. The poor-fit of the loose socket resulted in the generation of high pressures in pressure-sensitive areas, such as the distal-end of the stump. Then the sampled pressures from the loose socket simulation results were fed into the DSS, which in turn output a rectified socket design. The rectified socket was simulated and the new pressure distribution was compared with the nominal and loose socket simulations. The simulation was repeated for several variants produced by the fuzzy inference engine. The variants correspond to different thresholds of the pressure values: thresholds that result in high rectifications, medium rectifications and low rectifications. The results from each variant are compared and evaluated.

The sensor deployment on the socket and its relationship with anatomical regions are shown in Table A2. $z_{max}$ and $z_{min}$ are distances from the highest point of the proximal lateral brim. This point is also the basis for calculating the $\theta_{min}$ and $\theta_{max}$ angles clockwise (i.e., it is at 0 degrees). The pressure measurements of each sensor are the mean pressure values of the area defined by the coordinates ($z_{max},z_{min},\theta_{min},\theta_{max}$).

### 5.2. Results

**Figure 9:**
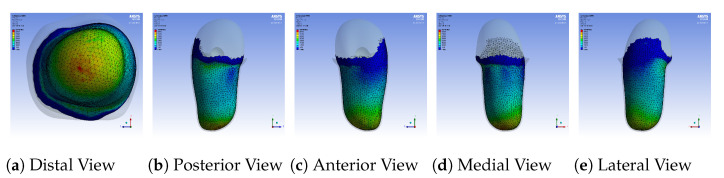
Pressure distribution over the entire stump after the donning process with the nominal socket.

**Figure 10:**
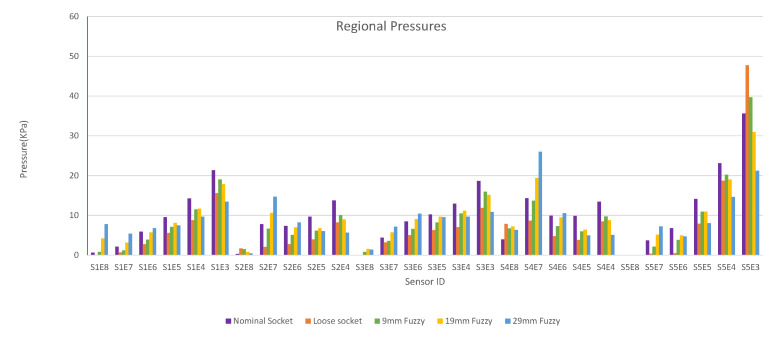
Regional pressure values for nominal, loose and rectified sockets.

A sample pressure distribution of the stump’s surface after the donning process with the nominal socket is shown in Figure 9. The pressure values of each sensor, after simulating the donning process for the nominal, loose and rectified sockets, are shown in Figure 10. The rectified socket is the design correction proposed by the DSS for the loose socket and has increased thickness on the antero-lateral front. The magnitude of this thickness has three different values: 9 mm (low), 19 mm (medium) and 29 mm (high), depending on threshold tuning.

The first observation worth nothing is that the loose socket generates high pressures at the distal-end. This was expected, as explained in Section 3.2.1, because the loose fit allows the stump to slide into the socket and wedge to the bottom, which in turn results in the increase of the pressures in the distal-end area. The pressures were normalized (according to the threshold of the variants) and were sent to the DSS, which outputs the rectification solution.

**Figure 11:**
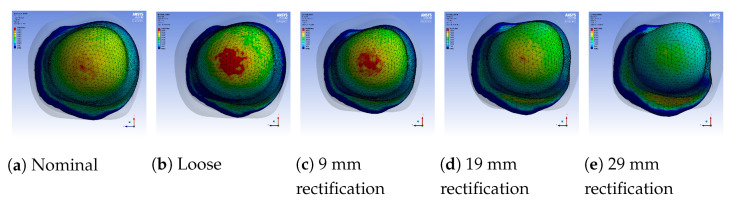
Regional pressure values in the distal-end region of the stump for different sockets.

**Figure 12:**
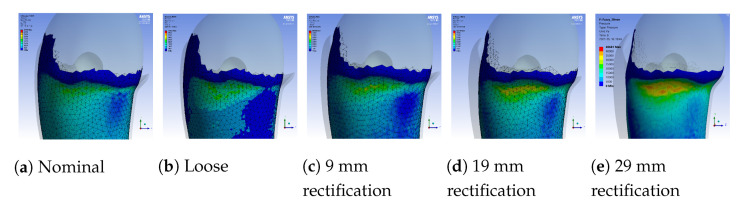
Regional pressure values in the proximal posterior region of the stump for different sockets.

Figure 10 shows that by increasing the thickness of the inner wall, the rectified sockets result in pressure profiles that tend to the pressure profile of the nominal socket. The following observations can be made:

1. The pressure experienced at the distal-end (S5E3) reduces as the rectification thickness increases.
2. The pressure distribution on the proximal areas (S1E8, S1E7, S1E6, S2E7, S2E6, S3E7, S3E6, S3E5, S4E7, S4E6, S5E7) becomes higher as the rectification increases.
3. As the thickness of the rectification increases, a trade-off is observed between offloading pressure from the distal end and increasing the pressure at the proximal areas of the stump. This can be easily seen in Figure 10, Figure 11 and Figure 12. As a result, medium thickness rectification provides the best rectification results, since it balances between offloading the pressure distribution on the distal end of the stump while maintaining relevantly low pressures on the proximal regions.

The above observations are explained by the fact that the rectification produced a tighter socket at the proximal and mid-regions. As a result the reduced volume held and fastened the stump tissue relatively high in the socket so that the distal-end did not push against the socket wall and no high pressures were exerted, as explained in Section 3.2.1. It should be noted that not all sensors made contact with the stump during donning. As a result, one or more sensors in the proximal region could detect zero pressures. For example, sensor S5E8 present near the proximal brim detected zero pressure, as seen in Figure 10.

## 6. Conclusions

A decision support system (DSS) for lower-limb prosthesis rectification has been developed. It is based on a fuzzy inference system. It accepts as input, pressure measurements generated by the SocketSense sensors embedded in the socket. The DSS applies rules that emulate the thought process of a prosthetist towards rectifying the socket. The output of the DSS is socket rectifications for improving socket fitness and patient comfort, which are demonstrated to the user (e.g., a prosthetist) in the form of a 3D CAD visualization.

The rectification results were tested in an FEA simulation environment where the donning process was simulated, and the pressures developed in the socket for different rectifications were demonstrated. Three different rectifications were tried by tuning the pressure thresholds of the rules. The results demonstrate how the inference engine can generate rectifications that result in pressure distribution improvement, i.e., reducing pressures at pressure-sensitive areas and increasing pressures at pressure tolerant areas of the stump, which corresponds to greater comfort. However as the rectification intensity increases, high pressures appear in other areas of the socket which results in discomfort. Thus, a trade-off emerges between the magnitude of the rectification and the balance of pressures experienced. The selected socket is the one that provides the best balance in this antagonistic relationship.

Future work includes (i) the enrichment of the set of static rules and the derivation of a set of dynamic rules; (ii) the verification of the results by conducting experiments with the actual SocketSense hardware and a patient; (iii) the comparison of the fuzzy logic inference engine with different AI algorithms, e.g., evolutionary algorithms such as monarch butterfly optimization (MBO) [27], elephant herding optimization (EHO) [28] and moth search (MS) [29]; and the precise calculation of the trade-off observed.

## Figures and Tables

**Table 1:** Material properties of bodies in ANSYS Mechanical Workbench.

Body	Material Properties
Femur	Young’s Modulus E=15 GPa,
	Poisson’s Ratio γ=0.3
Socket	Young’s Modulus E=1.5 GPa,
	Poisson’s Ratio γ=0.3
Stump	3 Parameter hyper-elastic (Mooney–Rivlin)
	$C_{10}=4.25$ KPa,
	$C_{11}=0$ KPa,
	$D_{1}=2.36$ MPa${}^{-1}$

## Data Availability

Not applicable.

## Appendix A

**Table A1:** Logistic function parameters.

θ Range	$x_{0}$	*L*	*k*	*n*
$\theta_{AS}\leq \theta \left(p_{i}\right)\leq \theta_{AS}+0.1\cdot \Delta \theta_{max}$	$\theta_{AS}+0.05\cdot \Delta \theta_{max}$	$m_{A}$	0.5	1
$\theta_{AL}-\frac{\Delta \theta_{max}}{2}\leq \theta \left(p_{i}\right)\leq \theta_{AL}+\frac{\Delta \theta_{max}}{2}$	$\theta_{AL}$	$max(m_{A},m_{L})$	0.5	$sign(m_{A}-m_{L})$
$\theta_{LE}-0.1\cdot \Delta \theta_{max}\leq \theta \left(p_{i}\right)\leq \theta_{LE}$	$\theta_{LE}-0.05\cdot \Delta \theta_{max}$	$m_{L}$	0.5	−1

**Table A2:** Mapping of sensors to regions.

Sensor id	$z_{\max}\left(\mathrm{mm}\right)$	$z_{\min}\left(\mathrm{mm}\right)$	$\theta_{\min}\left(\mathrm{Degrees}\right)$	$\theta_{\max}\left(\mathrm{Degrees}\right)$	Anatomical Region
S1E8	0	−50	0	30	Proximal Lateral
S1E7	−50	−100	0	30	Proximal Lateral
S1E6	−100	−150	0	30	Proximal Lateral
S1E5	−150	−200	0	30	Proximal Lateral
S1E4	−200	−250	0	30	Distal Lateral
S1E3	−250	−300	0	30	Distal Lateral
S2E8	−50	−100	−150	−120	Ramus
S2E7	−100	−150	−150	−120	Proximal Medial
S2E6	−150	−200	−150	−120	Proximal Medial
S2E5	−200	−250	−150	−120	Distal Medial
S2E4	−250	−300	−150	−120	Distal Medial
S3E8	0	−50	−30	0	Lateral Gluteal Fold
S3E7	−50	−100	−30	0	Lateral Gluteal Fold
S3E6	−100	−150	−30	0	Lateral Gluteal Fold
S3E5	−150	−200	−30	0	Proximal Posterior
S3E4	−200	−250	−30	0	Distal Posterior
S3E3	−250	−300	−30	0	Distal Posterior
S4E8	−50	−100	−120	−90	Ischium
S4E7	−100	−150	−120	−90	Proximal Adductor Magnus
S4E6	−150	−200	−120	−90	Proximal Adductor Magnus
S4E5	−200	−250	−120	−90	Distal Adductor Magnus
S4E4	−250	−300	−120	−90	Distal Adductor Magnus
S5E8	−50	−100	150	180	Scarpa’s Triangle
S5E7	−100	−150	120	150	Proximal Anterior
S5E6	−150	−200	90	120	Proximal Anterior
S5E5	−200	−250	60	90	Distal Anterior
S5E4	−250	−300	30	60	Distal Antero-lateral
S5E3	−335	−350	0	−90	Femur Relief
